# Vitamin E as an Adjuvant Treatment for Non-alcoholic Fatty Liver Disease in Adults: A Systematic Review of Randomized Controlled Trials

**DOI:** 10.7759/cureus.9018

**Published:** 2020-07-06

**Authors:** Muhammad Usman, Nabiyah Bakhtawar

**Affiliations:** 1 Internal Medicine, Leicester Royal Infirmary, Leicester, GBR; 2 Internal Medicine, Kettering General Hospital, Kettering, GBR; 3 Surgery, Lister Hospital, Stevenage, GBR

**Keywords:** nafld, vitamin e, non-alcoholic fatty liver disease, tocopherol, antioxidant, oxidative stress, systematic review

## Abstract

Nonalcoholic fatty liver disease (NAFLD) is one of the most common causes of chronic liver disease. It is characterized by a variety of pathologies, ranging from benign fatty liver to extensive fibrosis and even hepatocellular cancer. Among the several potential risk factors, insulin resistance and increased oxidative stress are the most important. Vitamin E is an antioxidant with a potential to be used as a treatment for NAFLD. Therefore, we carried out a structured systematic review of all RCTs conducted between 2010 and January 2020. After screening, eight RCTs were included. Our systematic review showed that vitamin E has clinical utility in improving biochemical (ALT and AST levels) and histological abnormalities in NAFLD (hepatic steatosis and lobular inflammation). However, vitamin E does not seem to have significant effects on liver fibrosis. Still, vitamin E has the potential to be used as an adjuvant for the treatment of NAFLD, and its use in clinical practice should be advocated.

## Introduction and background

Non-alcoholic fatty liver disease (NAFLD) is an umbrella term used to represent a variety of liver pathologies, ranging from benign non-alcoholic fatty liver (NAFL) to progressive non-alcoholic steatohepatitis (NASH) with or without fibrosis, NASH cirrhosis, and hepatocellular carcinoma (HCC) [1-2]. NAFLD is a diagnosis of exclusion and is characterized by ≥5% hepatic fat accumulation in the absence of a history of drinking and other causes of chronic liver disease such viral hepatitis, hemochromatosis, Wilson’s disease, autoimmune hepatitis, drug-induced hepatitis, and chronic hepatitis due to endocrine or hereditary causes [2].

The pathogenesis of NAFLD is poorly understood, but it is likely to be a multi-factorial, multi-step, and progressive disease. It is quite likely that metabolic syndrome remains the epicenter of the disease [2]. In addition to insulin resistance, increased production of reactive oxygen species (ROS), oxidative stress, and mitochondrial dysfunction are the key pathologies, leading to biochemical and histological derangements seen in NAFLD [3].

The risk factors established to play a role in the pathogenesis of this disease include central obesity, hyperglycemia, hypertriglyceridemia, and hypertension [1-4]. Other potential risk factors that are being investigated include genetic factors (*PNPLA3* and *TM6SF2* genes), dysbiosis of gut microbiota, dietary modifications (such as high fructose consumption), and endocrine issues (such as hypothyroidism, hypogonadism, and hypopituitarism) [2-4].

There has been a staggering increase in the number of NAFLD cases over the past 20 years. The prevalence varies according to the region and can be as low as 13.5% of all liver disease patients in Africa compared to 46% in America [5-6]. Furthermore, the disease prevalence can be as high as 55.5% in type II diabetics [7].

Lifestyle modifications are the first-line management for NAFLD. Among the pharmacological agents, pioglitazone and vitamin E have shown some promise [8]. Vitamin E is a potent antioxidant that improves biochemical and histological abnormalities associated with NAFLD in various studies [9-10].

Despite the potential benefits of vitamin E for the treatment of NAFLD and the randomized controlled trials (RCTs) conducted to validate this fact, there is a lack of systematic reviews that could help conclude these studies. There has been some work where authors have looked at the effects of vitamin E among all population groups [11]. However, there exists a great deal of disproportionation between the prevalence of NAFLD among different age groups, with the disease becoming more prevalent in the aging population [12].

There are a limited number of systematic reviews that specifically look into the effects of vitamin E on NAFLD outcomes for the adult population. Therefore, this systematic review aims to look at the therapeutic potential of vitamin E used as an adjuvant for NAFLD treatment in the adult population.

## Review

Methods

Information Sources

Two independent reviewers (UM, BN) carried out a rigorous literature review using electronic databases. The PRISMA guidelines for carrying out a systematic review were followed. PubMed, Google Scholar, Medline, EMBASE, CENTRAL, and clinical trial directories were searched for peer-reviewed randomized control trials conducted between 2010 and January 2020. Two search themes were used to search the databases that were combined using the Boolean operator ‘AND’. For the theme of ‘NAFLD’, we used keywords such as NAFLD, NASH, liver cirrhosis, and liver disease. For the theme ‘vitamin E’, we used keywords such as vitamin E, tocotrienol, and tocopherol.

Inclusion Criteria

The search was only limited to RCTs, full-text publications, articles published in English, and studies recruiting adult population (age ≥18). Trials that investigated the effects of vitamin E on biochemical markers (ALT, AST) and/or histological markers (steatosis, ballooning, inflammation, and fibrosis) were considered

Exclusion Criteria

The exclusion criteria included (1) all studies conducted on children and adolescent population, (2) studies involving individuals with liver disease due to alcohol or secondary causes such as viral hepatitis, hemochromatosis, Wilson’s disease, autoimmune hepatitis, drug-induced hepatitis, and chronic hepatitis due to endocrine or hereditary causes, and (3) studies that were not RCTs were excluded from the study.

Data Extraction and Study Selection

Both the researchers carried out an individual review of the literature. All results were compiled and both researchers compared their data and solved any conflicts through mutual concurrence and consultation.

A total of 345 studies were identified through a literature search. A total of 290 studies were excluded as they were in different languages, were not full-text studies, and because of their study design (included adolescents/children). The researchers read through 55 studies manually and excluded another 47 after reading through the article titles and abstracts. A total of 8 RCTs were included in the final analysis.

**Figure 1:**
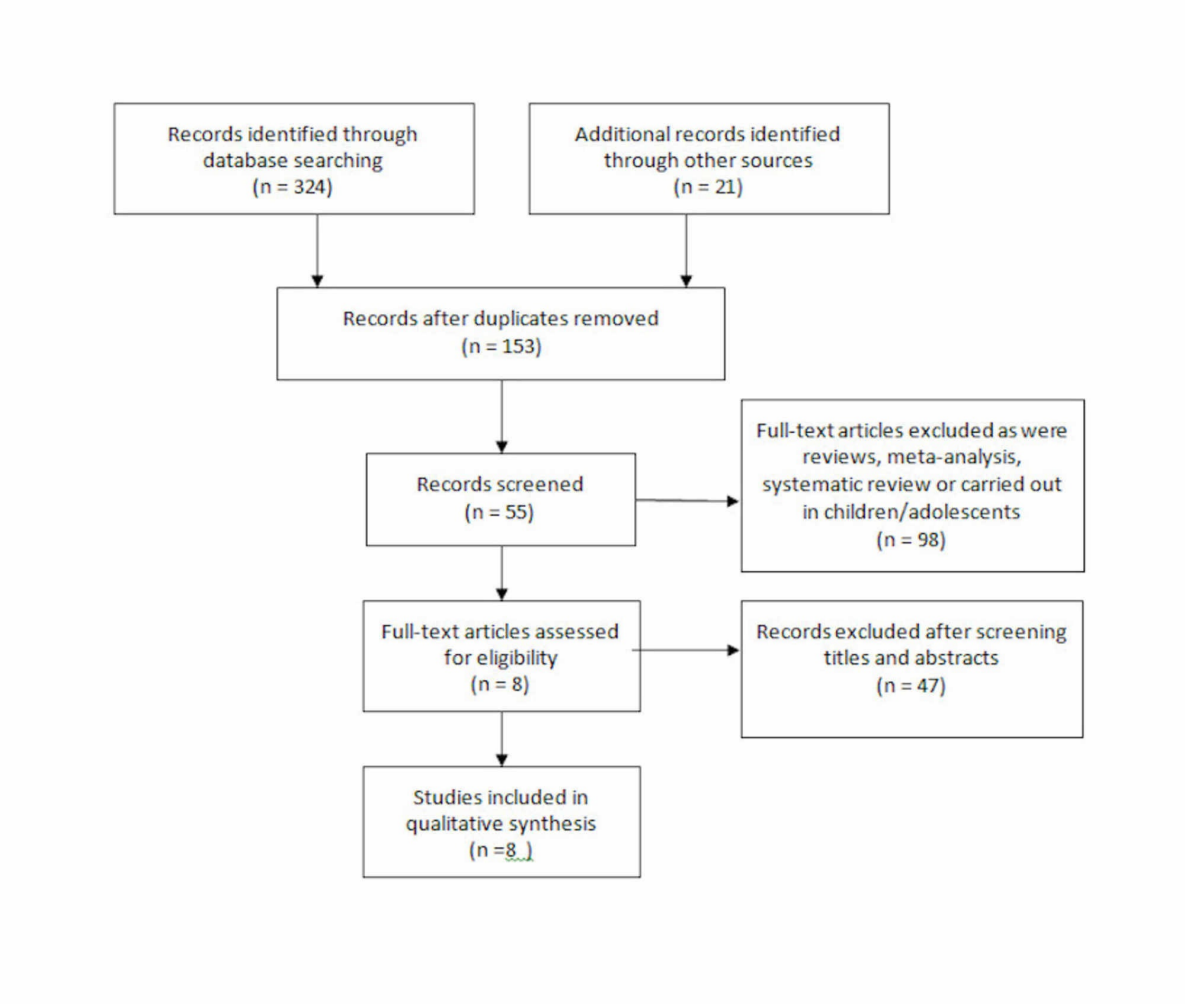
Flowchart based on PRISMA guidelines describing study identification and selection process

Figure 1 describes the literature review process.

Results

A total of eight RCTs published between 2010 and January 2020 were identified. All the trials included a total of 1,227 participants with the mean age ranging between 44.3 and 59 years. The duration of studies ranged from 3 months to 52 months. Table 1 includes the RCTs added in chronological order and summarizes the population demographics and interventions used.

**Table 1:** Summary of patient demographics, study design, and study interventions in the included RCTs RCT, randomized controlled trials [13-20]

Authors and year	Type of study	Duration of study	Sample size	Mean age	Gender	Interventions used
Sanyal et al. 2010	Three-arm RCT	2.5 years	n=247 Group 1 (pioglitazone):80 Group 2 (Vitamin E): 84 Group 3 (Placebo): 83	46.3	Females: 60% Males: 40%	Group 1: pioglitazone 30 mg once daily Group 2: Vitamin E 800 IU once daily Group 3: placebo
Foster et al. 2011	Double-blinded RCT	4 years	n=455 Group 1 (Atorvastatin, vitamin C, and vitamin E): 229 Group 2 (Placebo): 226	59	Females:29.1% Males: 70.9%	Group 1: atorvastatin 20 mg, vitamin C 1 g, and vitamin E 1,000 IU once daily Group 2: Placebo
Hoofnagle et al. 2013	Triple-arm RCT	2.5 years	n=139* Group 2 (Vitamin E treatment groups): 71 Placebo group: 68 *final number after exclusion	45.5	Females:57.5% Males: 42.5%	Group 1: Pioglitazone 30 mg and vitamin E placebo Group 2: Vitamin E 800 IU vitamin once daily and pioglitazone placebo Group 3: Both placebo
Aller et al, 2015	RCT	3 months	n= 36 Group 1 ( Eurosil 85®, MEDAS SL per day plus lifestyle modifications): 18 Group 2 (Lifestyle modifications alone): 18	47.4	Females: 38.9% Males: 61.1%	Group 1: 2 tablets of silymarin per day (Silybum marianum Gaerth, 540.3 mg) plus vitamin E (36 mg) (Eurosil 85®, MEDAS SL) plus dietary restriction and exercise Group 2: Lifestyle modifications alone
Polyzos et al. 2017	Double blinded RCT	52 months	n= 31 Group 1 (vitamin E alone): 17 Group 2 (spironolactone plus vitamin E): 12	Study included individuals ≥18 years)	Females: 74.2% Males: 25.8%	Group 1: Vitamin E 400 IU twice a day Group 2: Spironolactone 25 mg once a day plus vitamin E 400 IU twice a day
Pervez et al. 2018	Double-blinded triple-arm RCT	3 months	n= 64 Group 1 (δ-tocotrienol): 31 Group 2 (placebo): 33	44.3	Females: 54.7% Males: 45.3%	Group 1: δ-tocotrienol 300 mg twice daily Group 2: Placebo
Bril et al. 2019	Triple-arm RCT	18 months	n= 105 Group 1 (Vitamin E plus placebo): 36 Group 2 (Vitamin E plus pioglitazone): 37 Group 3 (Both placebo): 32	59	Female: 11.4% Males: 88.6%	Group 1: vitamin E 400 IU twice a day plus placebo Group 2: vitamin E 400 IU twice a day plus pioglitazone 30 mg/day increased after 2 months to 45 mg/day, Group 3: Both placebo
Anushiravani et al. 2019	Five-arm RCT	3 months	n= 150 Group 1 (lifestyle modifications plus placebo): 30 Group 2 (lifestyle modifications plus vitamin E): 30 Group 3 (lifestyle modifications plus pioglitazone): 30 Group 4 (lifestyle modifications plus metformin): 30 Group 5 (lifestyle modifications plus silymarin): 30	47	Females: 48.7% Males: 51.3%	Group 1: lifestyle modifications plus placebo Group 2: lifestyle modification plus vitamin E 400 IU once a day Group 3: lifestyle modifications plus pioglitazone 15 mg once a day Group 4: lifestyle modifications plus metformin 500 mg once a day Group 5: lifestyle modifications plus silymarin 140 mg once a day

The study by Aller et al. did not clearly define the randomization strategy and included a combination therapy of vitamin E and silymarin tested against lifestyle modifications [16]. One double-blinded RCT compared a combination therapy of atorvastatin, vitamin C, and vitamin E and placebo [14]; the other double-blinded study compared vitamin E monotherapy and vitamin E combination therapy with spironolactone [17], and another compared δ-tocotrienol monotherapy therapy and placebo [18]. Three studies were triple-arm double-blinded RCTs where at least one group was given vitamin E as a monotherapy [13,15,19]. Anushiravani *et al*. carried out a five-arm double-blinded RCT, where vitamin E monotherapy was used in one group [20].

All the RCTs used varying doses of vitamin E, ranging from 400 IU to 1,000 IU [13-15,17,19-20]. Aller *et al*. used 36 mg vitamin E daily [16], and Pervez *et al*. used 600 mg δ-tocotrienol (an isoform of vitamin E) daily [18].

Pioglitazone was the most common co-intervention used in four of the eight RCTs [13,15,19-20]. This was followed by silymarin in two [16,20], lifestyle modifications in two [16,20], vitamin C in one [14], atorvastatin in one [14], spironolactone in one [17], and metformin in one of the studies [20]. 

Table 2 summarizes the effects of vitamin E on biochemical and histological markers of NAFLD.

**Table 2:** Table summarizing the effects vitamin E on biochemical and histological markers of NAFLD NAFLD, nonalcoholic fatty liver disease; ALT, alanine aminotransferase; AST, aspartate aminotransferase; ALP, alkaline phosphatase; GGT, gamma-glutamyl transferase [13-20]

Author and year	Improvement in liver function tests (ALT, AST, ALP, GGT)	Improvement in NAFLD Activity Score (NAS)	Improvement in steatosis	Improvement in fibrosis score	Improvement in ballooning	Improvement in lobular inflammation
Sanyal et al. 2010	Significant reduction in mean AST, ALT, ALP, and GGT levels	Yes	Yes	No	Yes	Yes
Foster et al. 2011	No	Not recorded	Yes	Not recorded	Not recorded	Not recorded
Hoofnagle et al. 2013	Significant reduction in ALT only	No	Yes	No	Yes	Yes
Aller et al. 2015	Significant reduction in GGT only	Not recorded	Not recorded	Yes	Not recorded	Not recorded
Polyzos et al. 2017	No	Not recorded	No	No	Not recorded	Not recorded
Pervez et al. 2018	Significant reduction in mean AST and ALT in the treatment group	Not recorded	No	Not recorded	Not recorded	Yes
Bril et al. 2019	Significant reduction in mean AST and ALT in both treatment groups	Not recorded	Yes	No	No	No
Anushiravani et al. 2019	Significant reduction in mean AST and ALT in treatment groups	Not recorded	Not recorded	Not recorded	Not recorded	Not recorded
No. of results showing improvement	6	1	4	1	2	3
No. of results showing no improvement	2	1	2	4	1	1
No. of results not recorded	0	6	2	3	5	4

Six of the eight RCTs noted significant improvements in liver function tests [13,15-16,18-20]. Four studies noted a significant improvement in both ALT and AST [13,18-20]; one noted improvement in ALT only [15], and one reported improvement in GGT only [16]. Sanyal *et al*. reported additional improvements in ALP and GGT levels [13]. Foster *et al*. and Polyzos *et al*. did not report any improvements in liver function tests with vitamin E therapy [14-17].

When liver histological parameters were evaluated, vitamin E treatment groups showed only limited improvements. Four RCTs showed improvement in steatosis [13-15,19], and three showed improvement in inflammation [13,15,18]. Sanyal *et al*. and Hoofnagle *et al*. reported an improvement in hepatocyte ballooning [13,15]. Only Aller *et al*. showed improvement in fibrosis [16], and Sanyal *et al*. showed improvement in NAFLD Activity Score (NAS) [13].

However, it must be noted that several markers of hepatic histological response (such as NAS, hepatocyte ballooning, and hepatocyte inflammation) were not evaluated in several studies [14-20] (Table 2). 

Discussion

Insulin resistance, metabolic syndrome, and oxidative stress are the key pathologies in NAFLD. Lifestyle modifications consisting of diet, exercise, and weight loss are still considered the first-line treatments for NAFLD [21-22]. However, the attrition and dropout rates during lifestyle modification regimens can be as high as 40% and 50%, respectively [23]. According to the European Association for the Study of the Liver (EASL), pharmacological therapies should be considered in cases of progressive NAFLD where lifestyle modifications are producing inadequate results [24]. Vitamin E is being currently recommended for the treatment of NAFLD by the American Association for the Study of Liver Diseases (AASLD) and the National Institute for Health and Care Excellence (NICE), UK [25].

Mitochondrial dysfunction and endoplasmic reticulum stress lead to increased lipid peroxidation. This causes an imbalance in pro-oxidants and antioxidants leading to increased oxidative stress and liver dysfunction in NAFLD [3]. In the context of NAFLD, vitamin E exerts a variety of effects. It acts as an antioxidant and reduces reactive oxygen species; increases the antioxidants like glutathione and superoxide dismutase, and lowers the hepatic inflammation and fibrosis by reducing the expression of pro-apoptotic (Bax), TGF-β, COX-2, and MMP-2 genes [9].

Erhardt *et al*. 2011 noted that serum levels of vitamin E in NAFLD patients were lower than controls [26]. Our meta-analysis showed the potential of vitamin E in the treatment of NAFLD. Our study showed that vitamin E leads to significant improvement in liver function test derangements seen in NAFLD, especially in AST and ALT levels. Our study is consistent with the current literature were similar findings were noted in previous meta-analyses [2,9].

However, the effects of vitamin E on liver histological parameters in NAFLD patients have been reported inconsistently in the literature. Our analysis showed that vitamin E is only able to improve hepatic steatosis and hepatic inflammation. It does not significantly improve liver fibrosis or other histological markers of disease activity like hepatocyte ballooning. Different findings have been reported in the literature, where Xu *et al*. 2015 reported a significant improvement in all histological indicators of liver injury including liver fibrosis, lobular inflammation, ballooning, and steatosis with the use of vitamin E. However, that meta-analysis included only three RCTs [27]. However, our findings have been validated by subsequently done more comprehensive meta-analyses and systematic reviews showing that vitamin E has limited efficacy in improving hepatic fibrosis. It might have some benefit in improving steatosis and lobular inflammation [2,9].

Limitations

It must be noted that the RCTs included in our systematic review have some limitations. There was a lack of standardization in the dose, form, and frequency of vitamin E used in the RCTs. The sample sizes in the current RCTs were relatively small. Moreover, the RCTs did not look into one or more significant factors useful for monitoring disease progression in NAFLD.

## Conclusions

Vitamin E is a potent antioxidant useful for the management of NAFLD. Our systematic review suggests that vitamin E helps improve several biochemical and histological derangements in NAFLD. However, it does not seem to improve some important aspects of NAFLD disease pathology such as hepatic fibrosis. Still, vitamin E has a significant potential to improve biochemical and histological indicators of NAFLD, especially when used as an adjuvant to other therapies such as lifestyle modification and pharmacotherapies such as pioglitazone. Therefore, vitamin E should be more commonly used in clinical practice as an adjuvant to the mainstay treatments for the management of NAFLD.
